# Supplementary material for: Supramolecular Fractal Growth of Self-Assembled Fibrillar Networks
Source: Gels. 2021 Apr 14;7(2):46. doi: 10.3390/gels7020046 (PMC8167784; doi:10.3390/gels7020046)
Supplement: Supplementary file 1 [file gels-07-00046-s001.pdf]

Supplemental information for

Supramolecular Fractal Growth of Self-Assembled Fibrillar Networks

Pedram Nasr [1], Hannah Leung [1], France-Isabelle Auzanneau [2], and Michael A. Rogers [1,*]

[1] Department of Food Science, University of Guelph; Guelph N1G 2W1, ON, Canada; pnasr@uoguelph.ca (P.N.); hleung07@uoguelph.ca (H.L.)
[2] Department of Chemistry, University of Guelph; Guelph N1G 2W1, ON, Canada; fauzanne@uoguelph.ca
* Correspondence: mroger09@uoguelph.ca; Tel.: +11-519-824-4120 (ext. 54327)

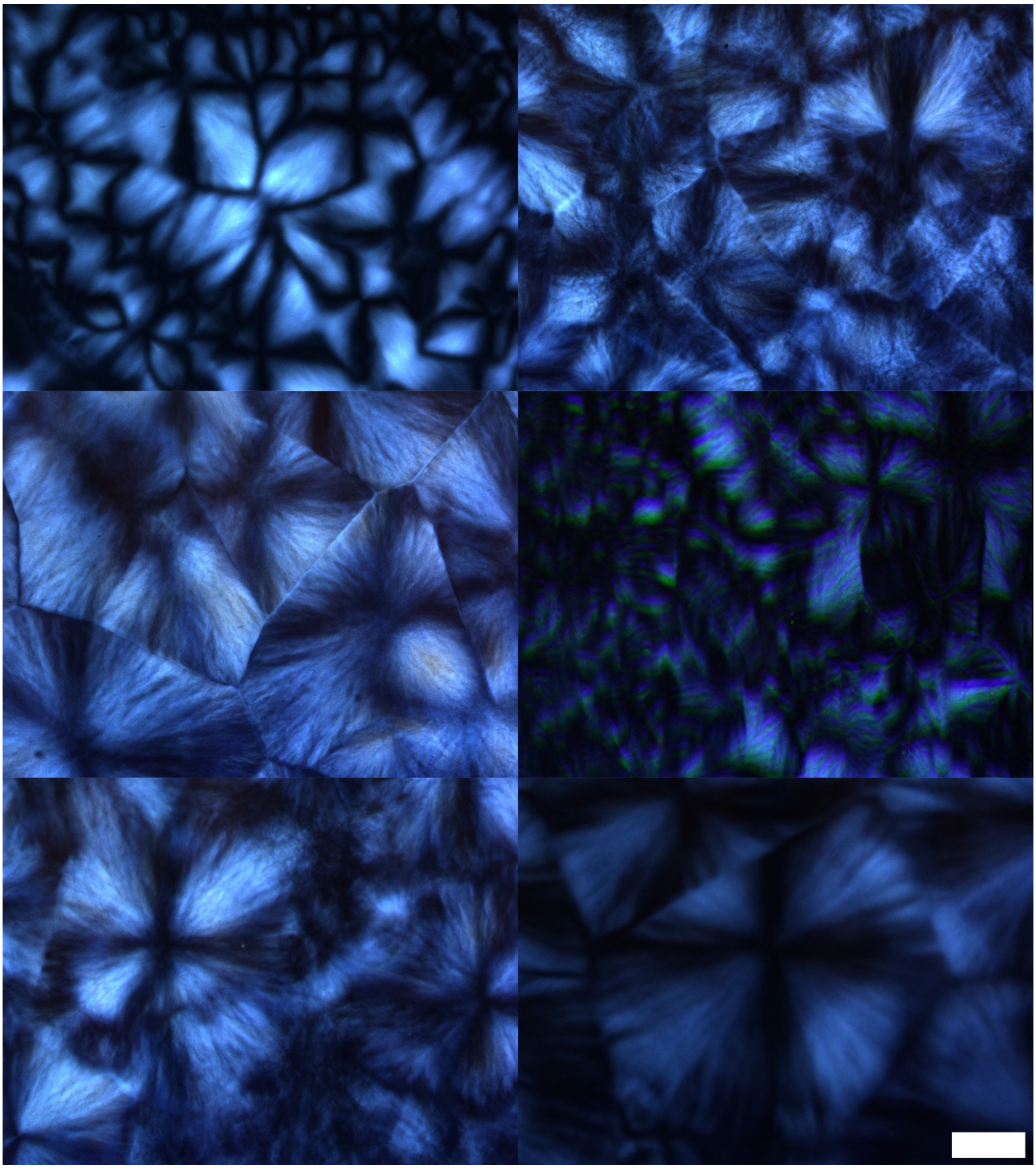

Figure S1 10x polarized light micrographs of 5wt% DBS in PEG200 crystallized in tripliciate. Scale bar = 100 μm.

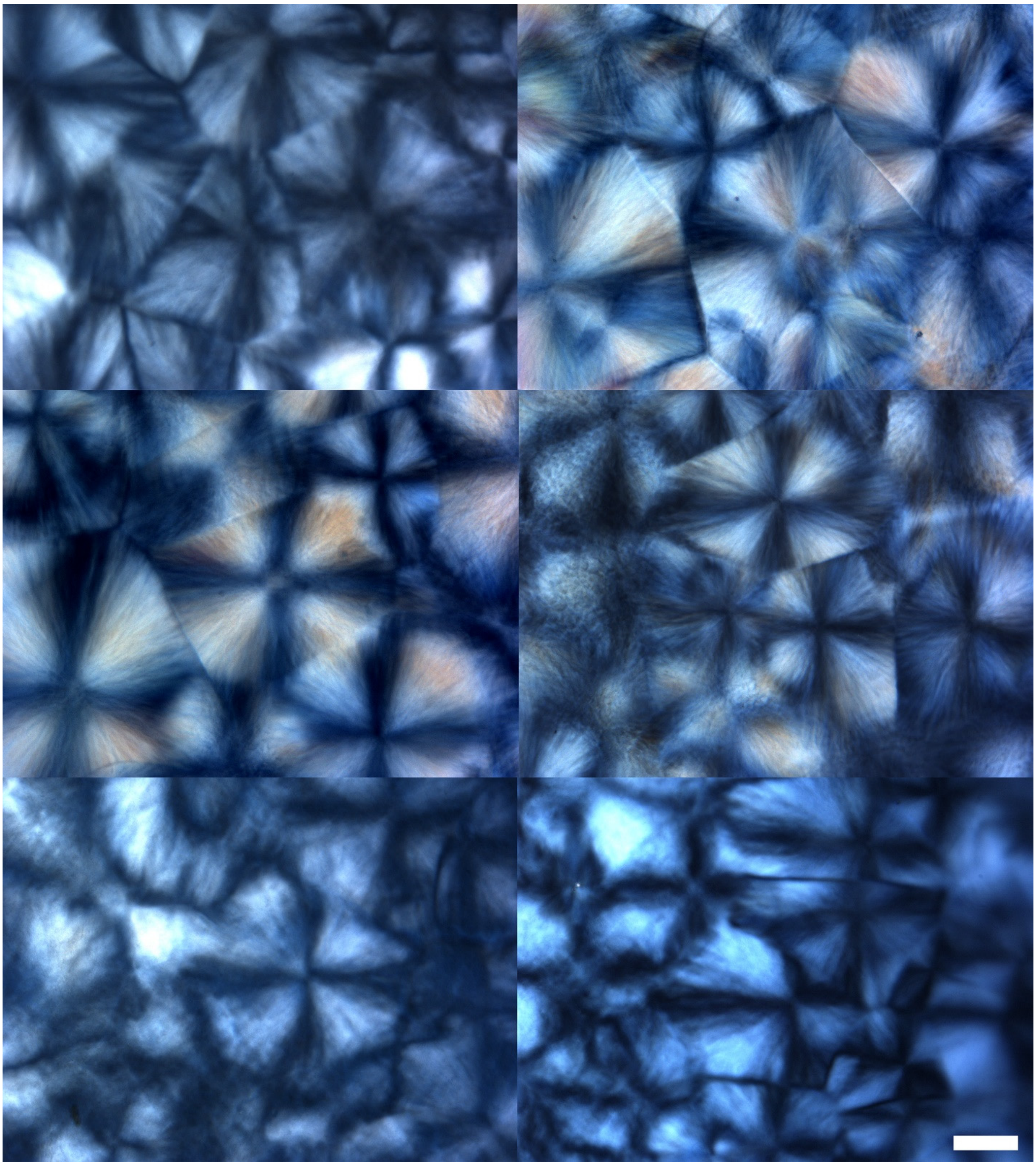

Figure S2 10× polarized light micrographs of 5wt% DBS in PEG600 crystallized in triplicate. Scale bar = 100 μm.

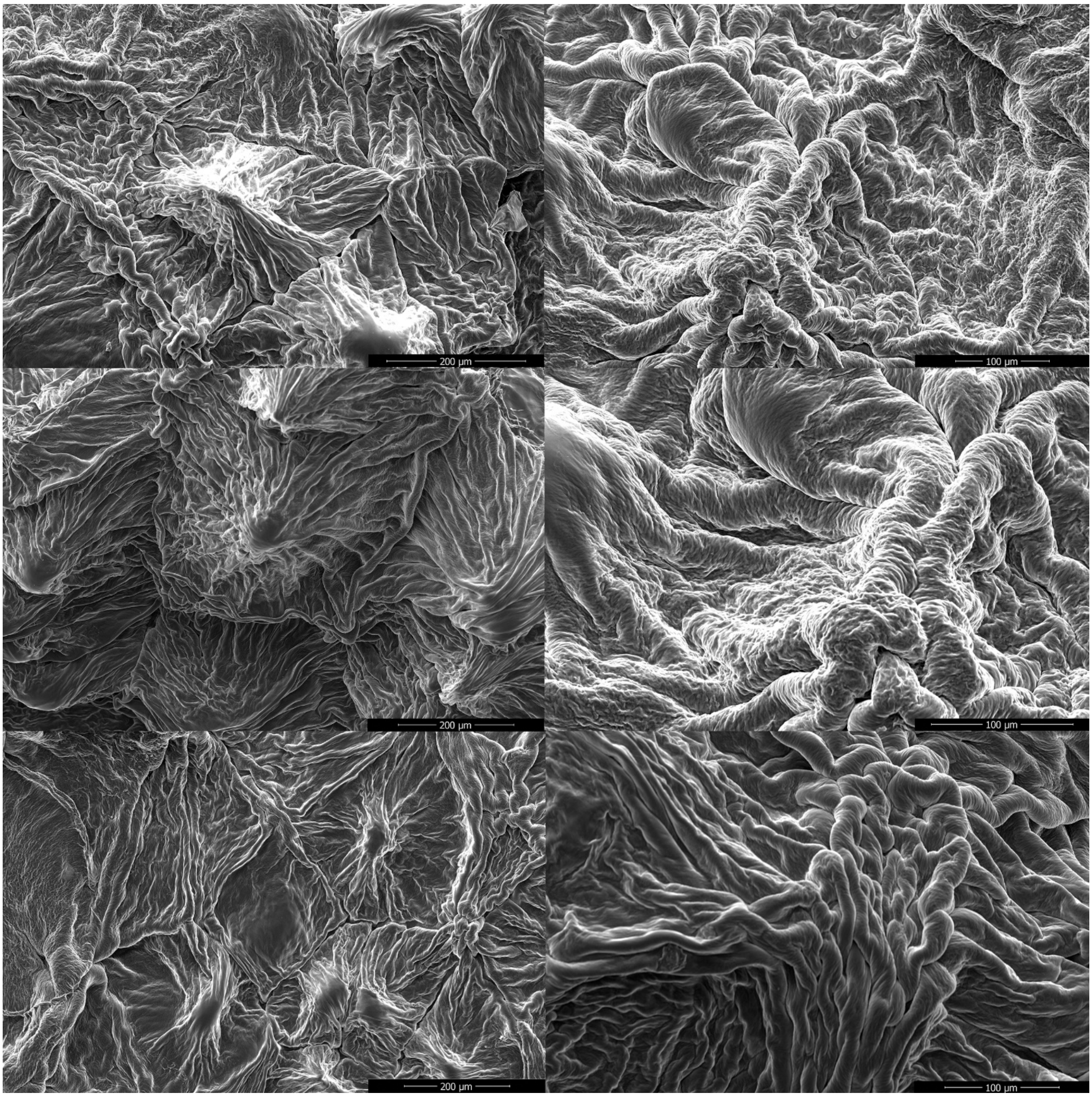

Figure S3 Scanning electron micrographs of 5wt% DBS in PEG200 crystallized on three separate SEM Spindles.

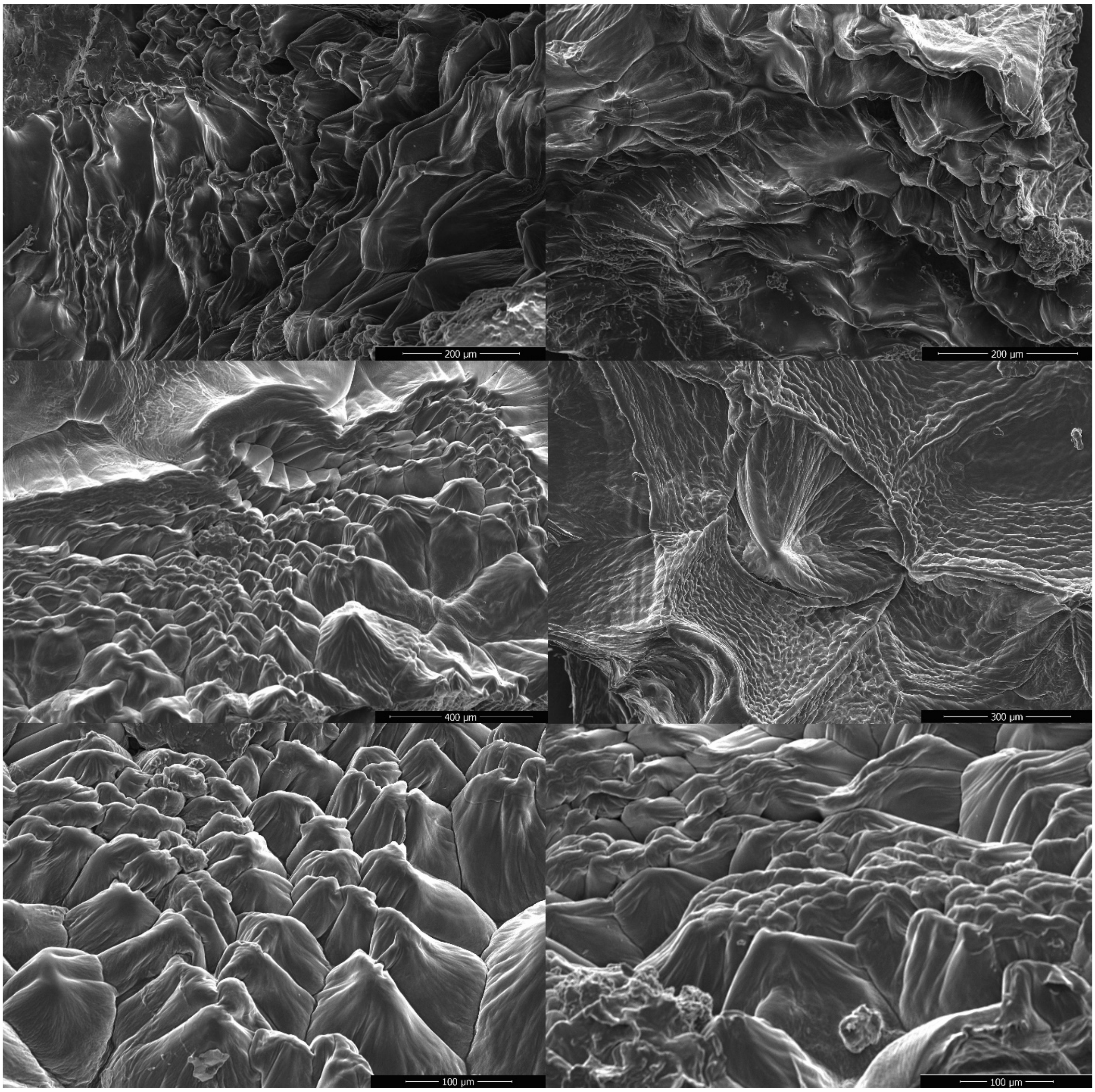

Figure S4 Scanning electron micrographs of 5wt% DBS in PEG600 crystallized on three separate SEM spindles.
